# Sulforaphane Ameliorates the Intestinal Injury in Necrotizing Enterocolitis by Regulating the PI3K/Akt/GSK-3*β* Signaling Pathway

**DOI:** 10.1155/2022/6529842

**Published:** 2022-05-11

**Authors:** Zhong-Kun Bao, Yan-Hong Mi, Xiao-Yu Xiong, Xin-Hong Wang

**Affiliations:** ^1^Department of Radiology, Women's Hospital, Zhejiang University School of Medicine, Hangzhou, China; ^2^Department of Radiology, The Children's Hospital, Zhejiang University School of Medicine, National Clinical Research Center for Child Health, Hangzhou, Zhejiang Province, China; ^3^Department of Neonatology, Women's Hospital, Zhejiang University School of Medicine, Hangzhou, Zhejiang Province, China; ^4^Department of Radiology, The Second Affiliated Hospital, Zhejiang University School of Medicine, Hangzhou, China

## Abstract

**Objective:**

Necrotizing enterocolitis (NEC) is a serious neonatal disease; this study aims to investigate the role of sulforaphane (SFN) in NEC-induced intestinal injury.

**Methods:**

An animal model of NEC was established in newborn mice and intragastrically administrated with SFN; then, the general status and survival of the mice were observed. H&E staining was used to observe the pathological changes of intestinal tissues. ELISA, immunohistochemical staining, and flow cytometry assays were used to detect the levels of inflammatory factors, including TNF-*α*, IL-6, and IL-17, the expression of Bax, Bcl-2, TLR4, and NF-*κ*B, and the percentages of the Th17 and Treg cells, respectively. GSK-3*β* expression levels were measured by immunofluorescence. IEC-6 and FHC cells were induced with LPS to mimic NEC in vitro and coincubated with SFN; then, the inflammatory factor levels and cell apoptosis rate were detected. Finally, Western blot was used to assess the expression of PI3K/Akt/GSK-3*β* pathway-related proteins in vitro and in vivo.

**Results:**

SFN improved the survival rate of NEC mice during modeling, alleviated the severity of the intestinal injury, and reduced the proportion of Th17/Treg cells. SFN could inhibit TLR4 and NF-*κ*B levels, decrease the release of inflammatory factors TNF-*α* and IL-6, suppress Bax expression, increase Bcl-2 expression, and inhibit apoptosis both in in vitro and in vivo models of NEC. Meanwhile, SFN regulated the expression of PI3K/Akt/GSK-3*β* pathway-related proteins in vitro and in vivo.

**Conclusion:**

SFN relieved the inflammatory response and apoptosis by regulating the PI3K/Akt/GSK-3*β* signaling pathway, thereby alleviating NEC in model mice and cells.

## 1. Background

Necrotizing enterocolitis (NEC) is an acquired disease that often occurs in neonates, especially in premature infants, which even exceeds 90% [1]. Approximately 1–8% of the neonates are hospitalized in the intensive care unit owing to NEC. The exact etiology of NEC is not clear, but increased permeability of the immunocompetent immature and immature intestine is a predisposing factor [2]. In addition, both breastfeeding and dysbacteriosis of the gut may induce NEC [3]. More and more studies have shown that intestinal bacteria are closely related to the pathogenesis of intestinal diseases, especially enteropathogenic *Escherichia coli* [4]. Enteropathogenic *Escherichia coli* is still the major causative factor for diarrhea in infants and children, causing significant rates of morbidity and mortality [5, 6]. It is capable of producing adhesion and causing localized lesions on the intestinal epithelium, disrupting the surfaces of the cells, thus leading to the effacement of the microvilli and disruption of the epithelial barrier function [6]. In addition to directly participating in the pathogenesis of NEC, intestinal bacteria affecting Th17/Treg immune balance through various pathways is also one of the important mechanisms of NEC [7].

As one subset of the CD4^+^T cells, Th17 cell plays a dual role in the pathogenesis of inflammatory bowel disease, which can not only protect the intestinal mucosa by maintaining the balance of the immune microenvironment but also aggravate the intestinal inflammatory response by proinflammatory cytokines [8]. Th17 cells can secrete IL-17, IL-21, and IL-22, as well as other inflammatory cytokines, such as IL-1, IL-6, IL-18, and TNF-*α* [9]. Among them, IL-17 is one of the important cytokines secreted by Th17 cells, which can induce inflammation and aggravate tissue injury [10]. Treg cell is another subset of the CD4^+^ T cells that plays a negative immunoregulatory role in the body and critically important in maintaining immune tolerance and immune balance [11]. Treg cells mainly secrete cytokines such as IL-4, IL-10, and TGF-*β* and are involved in a variety of immune diseases in the body [12]. Regulation of the Th17/Treg imbalance is important for the prevention and treatment of NEC, but the specific predisposing factors and mechanism of Th17/Treg imbalance are not clear at present.

The PI3K/Akt signaling pathway is involved in fundamental cellular processes including apoptosis, metabolism, cycle, and survival to play a significant role in cellular homeostasis as well as the body [13]. He et al. found that GSK-3*β* is an important substrate of Akt, and the activity of GSK-3*β* can be reduced by phosphorylated Akt, thereby inhibiting downstream inflammatory responses or apoptosis [14]. In previous studies, Miao et al. found that miR-151-5p could balance Th17/Treg by activating the PI3K/Akt signaling pathway [15]. Gao et al. found that yak milk-derived exosomes could alleviate lipopolysaccharide (LPS)-induced intestinal inflammation through modulation of the PI3K/AKT/C3 pathway [16]. Therefore, the PI3K/Akt/GSK-3*β* pathway is important in balancing Th17/Treg and intestinal protection.

Sulforaphane (SFN) is an organosulfur complex mostly abundant in Cruciferae. It is recognized as one of the natural products with remarkable cancer prevention and antioxidative activities [17]. Abundant studies about SFN have focused on it for the treatment of liver caner, colon cancer, breast cancer, prostate cancer, and other diseases [18]. SFN has been found to upregulate the expression of Nrf2, thereby inhibiting the release and expression of the inflammatory factors as well as the activation of NF-*κ*B [19]. Zhang and Wu found that SFN can protect against intestinal injury by regulating the inflammation, apoptosis, and oxidative stress in intestinal epithelial cells [20]. Xin et al. found that SFN could activate Nrf2 by stimulating the Akt/GSK-3*β*/Fyn pathway [21]. Al-Harbi et al. found that SFN could reduce Th17 immune response [22]. These findings suggest that SFN has potential in the treatment of intestinal diseases and may have a role in regulating the PI3K/Akt/GSK-3*β* pathway and Th17/Treg cells.

Therefore, we speculate that SFN has potential in modulating the PI3K/Akt/GSK-3*β* pathway to protect against the intestinal injury in NEC. In this experiment, through the establishment of the NEC model in vitro and in vivo, SFN was used for intervention to study the effect and mechanism of SFN on NEC treatment.

## 2. Materials and Methods

### 2.1. Grouping of Experimental Animals and Establishment of the Model

A total of 36 SPF grade C57BL/6 male mice aged 5–7 days were randomly divided into three groups: control group, necrotizing enterocolitis group (NEC group), and SFN + NEC group. The other two groups except the SFN group received saline for the first 11 days, and the mice in the SFN group received SFN (20 mg/kg/day by intragastric administration).

The NEC model was induced in the NEC group and SFN + NEC group from day 1 onwards. The NEC model was established by intragastric administration of hypertonic formula (50 *μ*l/g, 5 times a day, Similac Advance infant formula (Abbott Nutrition): Esbilac (PetAg) canine milk replacer, 2 : 1) on the one hand and hypoxia treatment (5% O_2_, 2 times a day, 10 min/time) on the other hand [23]. In addition, LPS (4 *μ*g/g/d) was required on days 2 and 3 of modeling. The general status and survival of neonatal mice were observed and recorded during the experiment. After 11 days, all survival mice were humanely killed with CO_2_, and blood and terminal ileum were collected for analysis.

### 2.2. H&E Staining and Pathological Scoring

Mouse intestinal tissues were fixed in 10% buffered formalin and histological sections were prepared, followed by assessment of H&E-stained intestinal sections. Two double-blind pathologists assessed the extent of damage to the ileum. A score of 0 represents completely normal architecture, 1 represents mild lesion with thin separation of the submucosa and/or lamina propria, 2 represents moderate lesion with separation of submucosa and/or lamina propria and submucosa and/or edema, 3 represents severe lesion with severe separation of the submucosa and/or lamina propria, submucosa and/or muscular layer is grossly edematous with focal villous sloughing, and 4 represents necrosis with villous loss and necrosis. The higher score of the two scientists was selected as the assessment result, and a score greater than or equal to 2 was NEC.

### 2.3. ELISA Test

The obtained blood was centrifuged for 15 min, and the supernatant was taken. The contents of MD-2 (Jiangsu Enzyme Immunoassay Industrial Co., Ltd., MM-44942M2), NF-*κ*B (MM-44802M1), TNF-*α* (MM-0122H2), IL-6 (MM-1011M2), CXCL1 (MM43835M2), IL-17 (MM-0170M2), and TLR4 (MM-43950M1) were measured according to the instructions of the ELISA kit. The final result was then read at 450 nm using an ELISA plate reader (MD, CMaxPlus).

### 2.4. Immunofluorescence

Mouse intestinal tissues were sectioned and deparaffinized to water and placed into a retrieval box containing EDTA antigen retrieval solution (Servicebio, G1206) for antigen retrieval. This is followed by serum blocking. They were incubated overnight (4°C) with primary antibody against GS3K*β* (Affinity, AF5016), followed by incubation with secondary antibody and DAPI for 120 min (37°C). They were observed under a fluorescence microscope, and GS3K*β* expression intensity was quantified by three investigators (blinded to the experiment).

### 2.5. Flow Cytometric Subpopulation

The connective tissue was removed after aseptic bowel harvesting, and the intestinal tissue was crushed and centrifuged (1000 rpm, 5 min). After removing the supernatant, 5 mL of red blood cell lysate was added for lysis for 5 min and centrifuged again. The supernatant was removed and PBS was added to prepare cells. 100 *μ*L of cells was used to add 2.5 *μ*L antibody of IL-17 (BD Biosciences Pharmingen, 560666), CD4 (562891), Foxp3 (560414), and CD25 (335807), respectively, and incubated for 30 min at 4°C in the dark. After incubation, the cells were resuspended and detected by flow cytometry.

### 2.6. Immunohistological Staining

Intestinal tissues were sectioned and deparaffinized into water, and 3% hydrogen peroxide solution (Sinopharm Chemical Reagent Co., Ltd., 100092683) was used for blocking endogenous peroxidase after antigen retrieval. Later, 3% BSA (Servicebio, G5001) blocking solution was added (60 min). The primary antibody to the target protein (TLR4 antibody (Affinity, AF7017), NF-kB p65 antibody (Affinity, AF5006), Bax antibody (Affinity, AF0120), and Bcl-2 antibody (Affinity, AF6139)) were incubated overnight at 4°C. Secondary antibody incubation with the corresponding species of the primary antibody was added the next day (37°C, 50 min). Then, DAB chromogenic solution was added, and the nuclei were counterstained with 100 *μ*l of hematoxylin, and the experimental results were observed under a microscope.

### 2.7. Cell Grouping and Treatment

Cultured rat small intestinal cells (IEC-6) and a human normal colorectal cell line (FHC) were obtained from the American Type Culture Collection (ATCC) and maintained in culture medium contained 10% fetal bovine serum (FBS) [24, 25]. We divided the cells into three groups, including the SFN + LPS group and LPS control group. Before modeling, the SFN + NEC group was pretreated with 2 *μ*m SFN for 12 h. After that, the control group was treated with 0.1% DMSO, and the NEC group was induced with LPS (6 h, 50 *μ*g/ml) to establish the NEC model.

### 2.8. Real-Time Quantitative PCR (qPCR)

Cells were lysed and total RNA was extracted using TRIzol reagent (Vitality, B511311). Reverse transcription was then performed to synthesize cDNA. The cDNA was then analyzed (SYBRPremixExTaq (TaKaRa)). The expression of target genes was calculated by the 2^−△△Ct^ method, and *β*-actin was used as an internal reference. Primer sequences of genes are given in Table 1.

### 2.9. Western Blot

Intestinal tissues from mice, IEC-6, and FHC cells in each group were subjected to protein extraction with RIPA lysis solution (Biyuntian, P0013D) for one hour at 4°C, respectively. Total protein was obtained after centrifugation. Proteins were aliquoted into 20 *μ*g protein aliquots, separated (10% SDS-polyacrylamide gel), and transferred to polyvinylidene fluoride (PVDF) membranes (GEHealthcareLife, 10600023). The corresponding primary antibody (anti-p-Akt antibody (Affinity, AF0016), anti-p-PI3K antibody (Affinity, AF3241), anti-P-GSK-3 antibody (Affinity, AF2016), anti-PI3K antibody (Affinity, AF6241), anti-GSK-3 antibody (Affinity, AF5016), anti-GAPDH antibody (Affinity, AF7021), anti-*β*-actin antibody (Affinity, AF7018), and anti-Akt antibody (Affinity, AF6261)) was incubated overnight at 4°C, and the corresponding secondary antibody (37°C, 120 min) was incubated. The signal is then visualized, and the fluorescence intensity of the signal was measured.

### 2.10. Flow Cytometry

500 *μ*L of binding buffer was added to each group of cells, respectively, and the supernatant was discarded after centrifugation. Sequentially add 5 *μ*L AnnexinV-FITC and 10 *μ*L PI and mix well. The reaction was performed for 15 min at room temperature in the dark. The apoptosis rate was measured by flow cytometry within 1 hour.

### 2.11. Statistical Analysis

All experimental data were analyzed and processed using SPSS 16.0 as well as the GraphpadPrism7 software. All data were expressed as mean ± standard deviation ($\bar{x}$ ± s), one-way ANOVA (one-way analysis of variance) was used for multiple groups of measurement data, and the SNK test was used for comparison. The Kruskal–Wallis H test was used for unequal or nonnormal variance.

## 3. Results

### 3.1. Effects of SFN on General Status, Survival Rate, and Intestinal Histopathological Changes in NEC Mice

**Figure 1:**
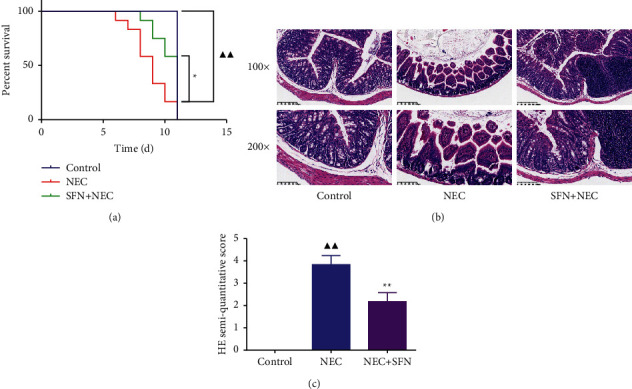
Effects of SFN on survival rate and intestinal histopathological changes in NEC mice. (a) Survival analysis of mice in each group. (b) H&E staining and semiquantitative scoring results of the intestinal tissues of mice in each group, *n* = 6. NEC, necrotizing enterocolitis; SFN, sulforaphane. Compared with the control group, ^▲^*P* < 0.05, ^▲▲^*P* < 0.01; compared with the NEC group, ^★^*P* < 0.05, ^★★^*P* < 0.01. Data represent the mean ± SD.

Through the observation of the general state of the mice, we found that compared with the mice in the control group, the activity of the NEC group was significantly less active, the mice responded more slowly, and there were urinary retention and abdominal distension phenomena. While the addition of SFN reversed the symptoms caused by NEC. We further analyzed the survival outcome of mice in each group, and the results are shown in Figure 1(a). Compared with the NEC group, the SFN + NEC group greatly improved the survival rate of mice (*P* < 0.05).

Observing the results of H&E staining (Figure 1(b)), we found that the intestinal tissue had clear layers and normal structure in the control group, and compared with the NEC group, there was significant invasion of the serosal layer and lesions, infiltration by inflammatory cells, and destruction of all epithelium and crypts, demonstrating that the NEC model was successfully established to some extent. There was again a significant reduction in the degree of disease in the SFN + NEC group compared with the NEC group. The results of pathological scoring further confirmed that SFN could significantly improve the NEC-induced intestinal histopathological changes (Figure 1(c)).

### 3.2. Effect of SFN on the Contents of NF-*κ*B, TLR4, TNF-*α*, IL-6, CXCL1, MD-2, and IL-17 in NEC Mice

**Figure 2:**
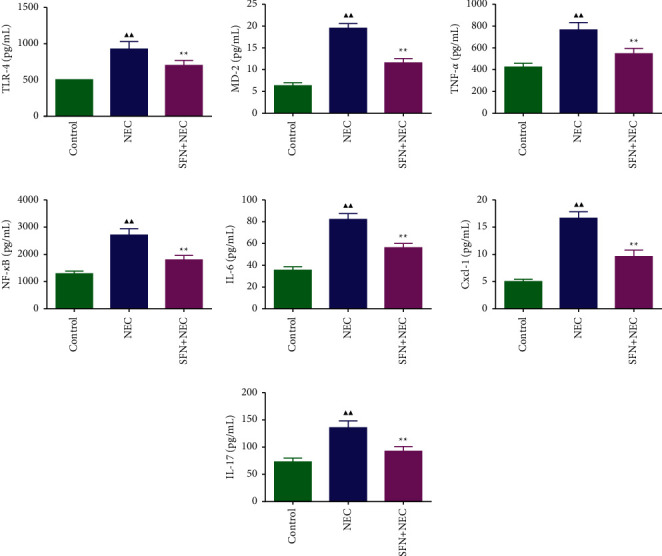
Changes of TLR4, MD-2, TNF-*α*, NF-*κ*B, IL-6, CXCL1, and IL-17 contents in the serum of NEC mice. NEC, necrotizing enterocolitis; SFN, sulforaphane. Compared with the control group, ^▲^*P* < 0.05, ^▲▲^*P* < 0.01; compared with the NEC group, ^★^*P* < 0.05, ^★★^*P* < 0.01. Data represent the mean ± SD, *n* = 6.

TLR4 and NF-*κ*B are important factors in the development of inflammatory response, and their signaling pathways can regulate the release of inflammation-related factors TNF-*α*, IL-6, IL-17, CXCL1, and MD-2. We used ELISA to detect the content of inflammation-related factors in the serum of NEC mice, and the results are shown in Figure 2. The contents of NF-*κ*B, TLR4, TNF-*α*, IL-6, IL-17, CXCL1, and MD-2 were significantly increased in NEC mice (*P* < 0.01). Changes in the content of inflammatory-related factors were reversed after treatment with SFN; SFN therefore significantly inhibits NEC -evoked inflammatory responses in vivo.

### 3.3. Changes in the Proportion of Th17 and Treg Cells in Intestinal Tissues of Mice

**Figure 3:**
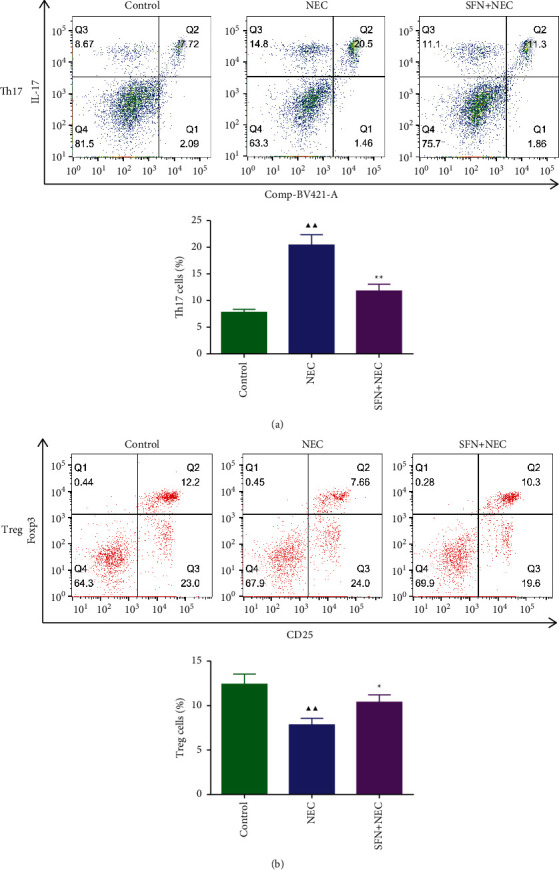
Changes in the proportion of Th17 and Treg cells in intestinal tissues of mice. NEC, necrotizing enterocolitis; SFN, sulforaphane. Compared with the control group, ^▲^*P* < 0.05, ^▲▲^*P* < 0.01; compared with the NEC group, ^★^*P* < 0.05, ^★★^*P* < 0.01. Data represent the mean ± SD, *n* = 3.

Th17/Treg cell imbalance promotes the development of NEC, in which Th17 cells have a proinflammatory effect and can induce apoptosis through the expression of the gene encoding IL-17A. We examined changes in the proportion of Th17 and Treg cells in mouse intestinal tissues. As shown in Figures 3(a) and 3(b), the percentage of Th17 was significantly increased and the percentage of Treg was significantly decreased in the NEC group compared with the control group (*P* < 0.05 or *P* < 0.01). SFN treatment reversed this result and therefore reduced the Th17/Treg cell ratio.

### 3.4. Expression Levels of TLR4, NF-*k*B, Bax, and Bcl-2 in Intestinal Tissues of Mice

**Figure 4:**
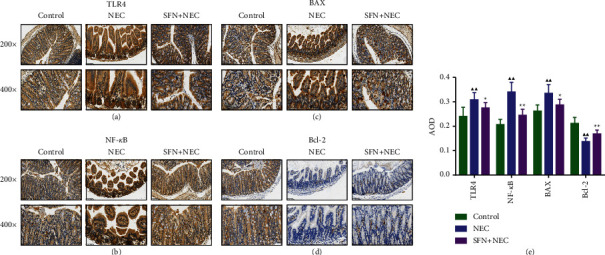
Immunohistochemical staining of TLR4 (a), NF-*κ*B (b), Bax (c), and Bcl-2 (d) and semiquantitative analysis of these proteins expression (e) in intestinal tissues of mice. NEC, necrotizing enterocolitis; SFN, sulforaphane. Compared with the control group, ^▲^*P* < 0.05, ^▲▲^*P* < 0.01; compared with the NEC group, ^★^*P* < 0.05, ^★★^*P* < 0.01. Data represent the mean ± SD, *n* = 6.

We performed immunohistochemical staining of mouse intestinal tissues to assess the expression levels of inflammatory pathway-related proteins TLR4, NF-*κ*B, and apoptosis-related proteins Bax and Bcl-2, and the results are shown in Figures 4(a)–4(d). Compared with the control group, the expression levels of TLR4, NF-*κ*B, and the proapoptotic protein Bax in the intestinal tissues of the NEC group were significantly increased (*P* < 0.01), and the expression of the antiapoptotic protein Bcl-2 was significantly decreased (*P* < 0.01). The results of protein expression reversed after treatment with SFN. Therefore, SFN could significantly inhibit the inflammatory pathway and decrease the Bax/Bcl-2 ratio in vivo.

### 3.5. Effect of SFN on the Expression Levels of PI3K/Akt/GSK-3*β* Pathway-Related Proteins in Intestinal Tissue of Mice

**Figure 5:**
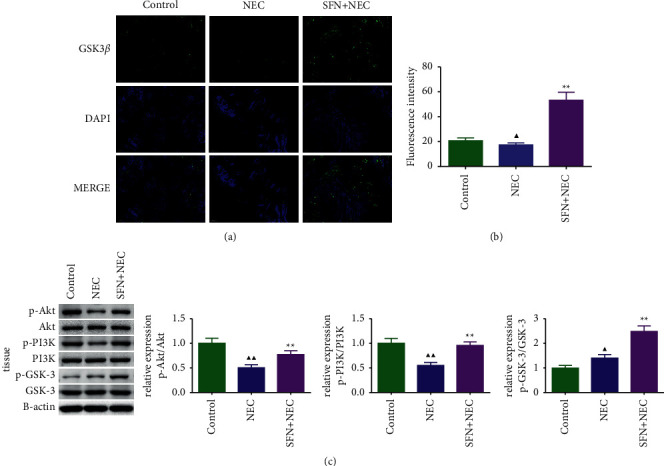
Expression levels of PI3K/Akt/GSK-3*β* pathway-related proteins in intestinal tissue of NEC mice. Immunofluorescence staining results of GSK-3*β* (a) and fluorescence intensity (b) in intestinal tissues of mice in each group, *n* = 6, and expression of PI3K/Akt/GSK-3*β* pathway-related proteins in intestinal tissue of mice, *n* = 3. NEC, necrotizing enterocolitis; SFN, sulforaphane. Compared with the control group, ^▲^*P* < 0.05, ^▲▲^*P* < 0.01; compared with the NEC group, ^★^*P* < 0.05, ^★★^*P* < 0.01. Data represent the mean ± SD.

We used immunofluorescence to detect GSK-3*β* expression levels in mouse intestinal tissues (Figures 5(a) and 5(b)) and found that SFN could significantly increase NEC-inhibited GSK-3*β* expression in vivo (*P* < 0.01). Then, we used Western blot to detect PI3K/Akt/GSK-3*β* pathway proteins expression levels, and the results are shown in Figure 5(c). We found that after treatment with SFN, the levels of p-Akt/Akt, p-PI3K/PI3K, and p-GSK-3/GSK-3 were significantly increased (*P* < 0.01).

### 3.6. The mRNA Expression Levels of TNF-*α*, IL-6, and IL-17 in FHC and IEC-6 Cells

**Figure 6:**
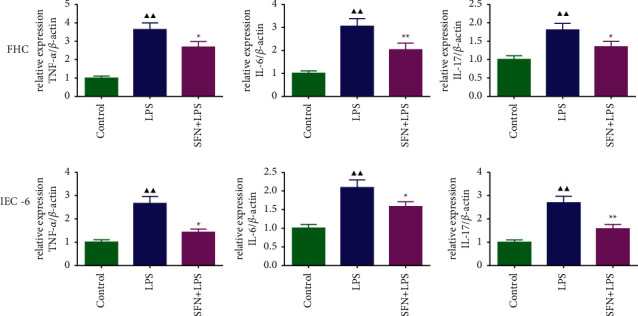
The mRNA expression of TNF-*α*, IL-6, and IL-17 in FHC and IEC-6 cells. LPS, lipopolysaccharide; SFN, sulforaphane. Compared with the control group, ^▲^*P* < 0.05, ^▲▲^*P* < 0.01; compared with the NEC group, ^★^*P* < 0.05, ^★★^*P* < 0.01. Data represent the mean ± SD, *n* = 3.

We detected the mRNA expression levels of TNF-*α*, IL-6, and IL-17 in FHC and IEC-6 cells, respectively, and the results are shown in Figure 6. The results showed that SFN could significantly reduce the mRNA expression levels of TNF-*α*, IL-6, and IL-17 in FHC and IEC-6 cells (*P* < 0.05 or *P* < 0.01). This demonstrated that SFN could inhibit the inflammatory response induced by NEC in vitro.

### 3.7. Effect of SFN on the Apoptosis of FHC and IEC-6 Cells

**Figure 7:**
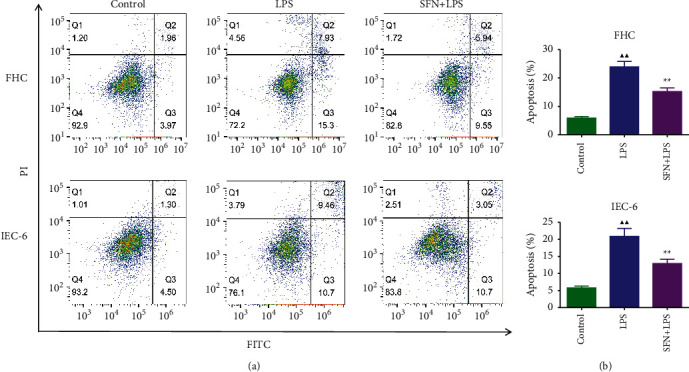
Apoptosis of FHC and IEC-6 cells. LPS, lipopolysaccharide; SFN, sulforaphane. Compared with the control group, ^▲^*P* < 0.05, ^▲▲^*P* < 0.01; compared with the NEC group, ^★^*P* < 0.05, ^★★^*P* < 0.01. Data represent the mean ± SD, *n* = 3.

The apoptosis of FHC and IEC-6 cells was detected by flow cytometry, and the results are shown in Figures 7(a) and 7(b). We found that SFN significantly inhibited the LPS-induced apoptosis in both cells (*P* < 0.01).

### 3.8. Expression Levels of PI3K/Akt/GSK-3*β* Pathway-Related Proteins in FHC and IEC-6 Cells

**Figure 8:**
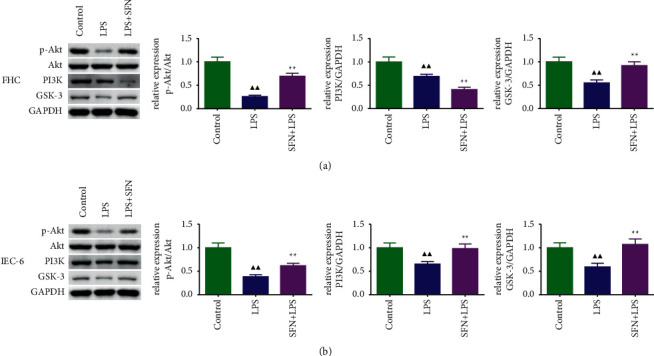
Expression of PI3K/Akt/GSK-3*β* pathway-related proteins in FHC (a) and IEC-6 (b) cells. LPS, lipopolysaccharide; SFN, sulforaphane. Compared with the control group, ^▲^*P* < 0.05, ^▲▲^*P* < 0.01; compared with the NEC group, ^★^*P* < 0.05, ^★★^*P* < 0.01. Data represent the mean ± SD, *n* = 3.

We examined the effect of SFN on PI3K/Akt/GSK-3*β* pathway proteins in vitro, and the results are shown in Figures 8(a) and 8(b). We found that after treatment with SFN, the expression of p-Akt and GSK-3 was significantly increased in both FHC and IEC-6 cells (*P* < 0.01), PI3K expression was significantly decreased in FHC cells (*P* < 0.01), and PI3K expression was significantly increased in IEC-6 cells (*P* < 0.01). Therefore, the PI3K/Akt/GSK-3*β* pathway is activated by SFN. However, PI3K expression in FHC cells decreased after SFN treatment, which has no obvious explanation. Therefore, our group will continue to focus on understanding the reasons for this.

## 4. Discussion

NEC is an acquired disease that often occurs in premature infants and is characterized by intestinal necrosis of mucosa or involved range [26]. Some studies have suggested that the imbalance caused by decreased Treg cells and increased Th17 cells is a key factor in NEC. SFN is an isothiocyanate with various effects such as regulating inflammation and inhibiting apoptosis [27]. Our study confirmed that SFN may prevent the imbalance of Th17/Treg cells by inhibiting the PI3K/AKt/GSK-3*β* signaling pathway, thereby alleviating intestinal injury to protect against NEC.

Numerous studies have shown that inflammation and apoptosis play an important role in the development of NEC [28, 29]. It has been found that the inhibition of intestinal inflammation is effective in reducing the incidence of NEC [30]. In the study by Hackam et al., it was found that when TLR4 was activated; it could activate the NF-*κ*B inflammatory pathway, trigger a series of inflammatory responses, promote inflammation in vivo, and trigger intestinal barrier failure, thereby aggravating NEC symptoms [31]. In addition, it has been found that the apoptosis of intestinal epithelial cells can lead to NEC, and the inhibition of apoptosis can inhibit ER stress, thereby alleviating intestinal injury [32]. Yang et al. suggested that NEC symptoms could be reduced by protecting intestinal apoptosis and inhibiting cellular inflammation [33]. Therefore, we speculated that SFN may have an ameliorative effect on NEC intestinal injury and that this may be related to the inhibition of inflammatory response and apoptosis. Based on the results in this study, we found that SFN had a protective effect on NEC by improving the survival of mice and alleviating histopathological injury of the intestine. In addition, our in vitro and in vivo findings showed that SFN can inhibit the activation of the TLR4/NF-*κ*B pathway, thereby inhibiting the transcription and release of inflammatory factors such as TNF-*α* and IL-6, and reducing the Bax/Bcl-2 ratio and inhibiting intestinal apoptosis. The results confirmed that SFN has a significant protective effect on the intestine of NEC and can ameliorate NEC-induced inflammation and apoptosis, which is consistent with previous studies.

Some hypotheses suggest that NEC results from an interplay between immature innate intestinal immune responses and the adverse microbial colonization [34]. Probiotics is reported with significant prophylactic and therapeutic potential in NEC, which can regulate the mucosal immune system, the gut microbiota, and the production of active metabolites such as short-chain fatty acids [35, 36]. Some previous reports have shown that SFN can affect the differentiation of Th17 and Treg cells. In a mouse model of asthma with mixed granulocytes, SFN could decrease IL-17 expression and Th17 infiltration [22]. Numerous previous studies have confirmed that Th17/Treg imbalance has an important role in the development of NEC [7]. Immune dysregulation in NEC is caused based on elevated Th17 and reduced Treg cell activity, and there is a significant positive correlation between Th17/Treg cell ratio and NEC severity [37]. With the development of NEC, the attention on Th17 cells continues to increase, as well as serum IL-17 levels and cytokine levels associated with Th17 cell differentiation [38]. At the same time, the proportion of Treg cells in the body decreases, resulting in the inability to exert a highly potent immunosuppressive function and ultimately causing intestinal mucosal injury to induce NEC [39]. We observed that Th17 was significantly increased, and Treg was significantly decreased in NEC mice, while SFN could effectively reverse this change, which was inhibited with these studies. This implied that SFN may suppress the function of Treg cells and increase the number of Th17 cells under immunosuppressive conditions, which contributes to its protective effect on NEC.

PI3K/AKt is associated with the differentiation and function of CD4^+^ T cells. In POF mice, after hPMSC transplantation, the PI3K/Akt signaling pathway can be involved in the recovery of ovarian function by altering the ratio of Th17/Tc17 and Th17/Treg cells [40]. In the context of Treg/Th17 imbalance induced by perinatal exposure to BPA in male offspring mice, the PI3K/Akt/mTOR signaling pathway can regulate Treg/Th17 ratio [41]. Our study observed that SFN could inhibit PI3K/AKt and decrease GSK-3*β* expression, consistent with these studies. Our data suggest that the PI3K/AKt/GSK-3*β* pathway responds to SFN and ultimately attenuates intestinal inflammation by modulating the Th17/Treg balance. However, the lack of corresponding experiments in this study to verify whether Akt exerts a feedback effect on the GSK-3*β* pathway in the context of NEC remains to be determined, and we will investigate this in further studies in the future.

## 5. Conclusion

In conclusion, this study explored the therapeutic potential of SFN for NEC and demonstrated that SFN may inhibit the inflammatory response and apoptosis by activating the PI3K/AKt/GSK-3*β* pathway to protect against intestinal injury induced by NEC. These results suggest that SFN may be used as a potential agent for the treatment of NEC.

## Figures and Tables

**Table 1:** qPCR primers sequences.

Gene	Forward primer	Reverse primer
Mouse *β*-actin	CCACAGCTGAGAGGGAAATC	TCTCCAGGGAGGAAGAGGAT
Mouse IL-6	CCAATTTCCAATGCTCTCCT	ACCACAGTGAGGAATGTCCA
Mouse TNF-*α*	CTCAAAACTCGAGTGACAAGC	CCGTGATGTCTAAGTACTTGG
Mouse IL-17	TGAAAACACAGAAGTAACGTCCG	CCCAGGAGGAAATTGTAATGGGA

## Data Availability

The dataset used and analyzed to support this study are available from the corresponding author upon request.
